# Direct ethanol production from cellulosic materials using a diploid strain of *Saccharomyces cerevisiae *with optimized cellulase expression

**DOI:** 10.1186/1754-6834-4-8

**Published:** 2011-04-15

**Authors:** Ryosuke Yamada, Naho Taniguchi, Tsutomu Tanaka, Chiaki Ogino, Hideki Fukuda, Akihiko Kondo

**Affiliations:** 1 Department of Chemical Science and Engineering, Graduate School of Engineering, Kobe University, 1-1 Rokkodaicho, Nada-ku, Kobe, Hyogo 657-8501, Japan; 2 Organization of Advanced Science and Technology, Kobe University, 1-1 Rokkodaicho, Nada-ku, Kobe, Hyogo 657-8501, Japan

## Abstract

**Background:**

Hydrolysis of cellulose requires the action of the cellulolytic enzymes endoglucanase, cellobiohydrolase and β-glucosidase. The expression ratios and synergetic effects of these enzymes significantly influence the extent and specific rate of cellulose degradation. In this study, using our previously developed method to optimize cellulase-expression levels in yeast, we constructed a diploid *Saccharomyces cerevisiae *strain optimized for expression of cellulolytic enzymes, and attempted to improve the cellulose-degradation activity and enable direct ethanol production from rice straw, one of the most abundant sources of lignocellulosic biomass.

**Results:**

The engineered diploid strain, which contained multiple copies of three cellulase genes integrated into its genome, was precultured in molasses medium (381.4 mU/g wet cell), and displayed approximately six-fold higher phosphoric acid swollen cellulose (PASC) degradation activity than the parent haploid strain (63.5 mU/g wet cell). When used to ferment PASC, the diploid strain produced 7.6 g/l ethanol in 72 hours, with an ethanol yield that achieved 75% of the theoretical value, and also produced 7.5 g/l ethanol from pretreated rice straw in 72 hours.

**Conclusions:**

We have developed diploid yeast strain optimized for expression of cellulolytic enzymes, which is capable of directly fermenting from cellulosic materials. Although this is a proof-of-concept study, it is to our knowledge, the first report of ethanol production from agricultural waste biomass using cellulolytic enzyme-expressing yeast without the addition of exogenous enzymes. Our results suggest that combining multigene expression optimization and diploidization in yeast is a promising approach for enhancing ethanol production from various types of lignocellulosic biomass.

## Background

Dwindling supplies of petroleum and growing environmental concerns over its use has led to increasing interest in developing biomass as a feedstock for liquid fuels. In particular, bioethanol produced from biomass represents a promising alternative fuel or gasoline extender. Currently, the main feedstock for bioethanol production is starch-rich biomass, as it is rapidly hydrolyzed by amylases, giving high yields of glucose. However, lignocellulosic biomass (such as rice straw, which is one of the most abundant lignocellulosic waste materials), is regarded as a promising starting material for bioethanol production, because it is abundant, inexpensive, renewable and has favorable environmental properties [1]. Despite these advantages, lignocellulosic biomass is much more expensive to process than grains because of the need for extensive pretreatment and relatively large amounts of cellulases for efficient hydrolysis [1]. Therefore, efficient and cost-effective methods for the degradation and fermentation of lignocellulosic biomass to ethanol are required.

The efficient degradation of lignocellulosic biomass requires the synergistic action of the cellulolytic enzymes endoglucanase (EG), cellobiohydrolase (CBH) and β-glucosidase (BGL), and some hemicellulolytic enzymes. Although there are numerous reports of lower-cost ethanol production from cellulosic material by consolidating hydrolyzing and fermentation steps using recombinant *Saccharomyces cerevisiae *strains expressing cellulolytic enzymes [2-5], the efficiency of cellulose degradation has not been sufficiently improved. Several filamentous fungi capable of effective cellulose degradation have also been identified (including *Trichoderma reesei*), which produce various cellulolytic enzymes, and simultaneously control their expression levels in response to their environment. The various cellulase proteins interact synergistically, and it is important that the ratios of the cellulases are appropriately balanced to achieve the maximum hydrolysis rate for a given amount of added cellulases [6,7].

We previously developed a simple method, named cocktail δ-integration, to optimize cellulase-expression levels for cellulose degradation [8]. In cocktail δ-integration, several kinds of cellulase-expression cassettes are integrated into yeast chromosomes simultaneously in one step, and strains with high cellulolytic activity (that is, expressing the optimum ratio of cellulases) can be easily obtained. Using this method, the phosphoric acid swollen cellulose (PASC) degradation activity of cellulase-displaying *S. cerevisiae*, which is a promising microorganism for efficient ethanol production from cellulose [9], significantly improved [8].

One of the advantages of our expression-optimization method is that the optimization process, which is based only on the target substrate-degrading phenotype and the target substrate itself can be easily altered. Thus, optimization of cellulase-expression levels for cellulose degradation can be achieved without prior knowledge of the optimum ratios of the target enzymes. In addition, genes integrated into the yeast genome by cocktail δ-integration are maintained stably in non-defined inexpensive industrial media, such as molasses-based and corn steep liquor (CSL)-based media [10,11].

Diploidization is another promising strategy to improve expression levels of heterologous genes and enhance the fermentation ability of *S. cerevisiae *[12-15]. Because polyploid yeast strains, including diploid strains, have higher cell growth rates, cell yields and tolerances to various stresses compared with haploid strains, they are particularly suited for industrial applications [14,16]. In a previous study, we developed a recombinant *S. cerevisiae *strain capable of efficient direct ethanol production from starch by combining the genome integration of amylase genes and diploidization [12,13]. Using this strategy, a diploid yeast strain was successfully constructed, which had enhanced amylase activity and growth rates compared with the initial haploid strains. However, the optimization of cellulase-expression ratios in a diploid yeast strain has not been reported, and may be an effective approach for improving ethanol fermentation.

In this study, the cocktail δ-integration method was used to optimize cellulase expression in two yeast strains of opposite mating types. These strains were mated to produce a diploid strain with enhanced cellulase expression, which was then evaluated for its efficiency in converting cellulose to ethanol from PASC and pretreated rice straw.

## Methods

### Strains, plasmids and media

Table 1 summarizes the genetic properties of the strains and plasmids used in this study. Briefly, the host for recombinant DNA manipulations was *Escherichia coli *strain NovaBlue (Novagen, Madison, WI, USA), and cellulolytic enzymes were expressed in the haploid yeast strains *S. cerevisiae *MT8-1 [17] and NBRC1440ΔHUWL [13]. The haploid and diploid *S. cerevisiae *strains 1440/cocδBEC3 and MNII/cocδBEC3, respectively, were constructed as described below.

**Table 1:** Characteristics of the bacterial and yeast strains and plasmids used in this study

Strains or plasmids	Relevant features	Reference
Bacterial strain		
*E. coli *Novablue	*endA1 hsdR17(r *_*K12*_^-^*m _K12_*^+^) *supE44 thi-I gyrA96 relA1 lac recA1/F*'[*proAB*^+ ^*lacI*^q ^ZΔM15::Tn*10*(Tet^r^)]	Novagen
*S. cerevisiae *yeast strains		
MT8-1	*MAT*a *ade his3 leu2 trp1 ura3*	[17]
NBRC1440ΔHUWL	*MATα his3 leu2 trp1 ura3*	[13]
MT8-1/IBEC	*MATa ade leu2*, Single copy Integration of β-glucosidase, endoglucanase and cellobiohydrolase gene	[8]
MT8-1/cocδBEC3	*MATa ade leu2*, Cocktail δ-Integration of β-glucosidase, endoglucanase and cellobiohydrolase gene	[8]
MT8-1/cocδBEC3/LEU2	*MATa ade*, Cocktail δ-Integration of β-glucosidase, endoglucanase and cellobiohydrolase gene	This study
1440/cocδBEC3	*MATα leu2*, Cocktail δ-Integration of β-glucosidase, endoglucanase and cellobiohydrolase gene	This study
MNII/cocδBEC3	*MATa/α*, Cocktail δ-Integration of β-glucosidase, endoglucanase and cellobiohydrolase gene	This study
Plasmids		
pδW-PGAGBGL	*TRP1*, Expression of β-glucosidase by δ-integration	[8]
pδU-PGAGBGL	*URA3*, Expression of β-glucosidase by δ-integration	[8]
pδH-PGAGBGL	*HIS3*, Expression of β-glucosidase by δ-integration	[8]
pδW-PGAGEG	*TRP1*, Expression endoglucanase by δ-integration	[8]
pδU-PGAGEG	*URA3*, Expression endoglucanase by δ-integration	[8]
pδH-PGAGEG	*HIS3*, Expression endoglucanase by δ-integration	[8]
pδW-PGAGCBH	*TRP1*, Expression cellobiohydrolase by δ-integration	[8]
pδU-PGAGCBH	*URA3*, Expression cellobiohydrolase by δ-integration	[8]
pδH-PGAGCBH	*HIS3*, Expression cellobiohydrolase by δ-integration	[8]
pRS405	*LEU2*, no expression	Stratagene

*E. coli *transformants were grown in Luria-Bertani medium (10 g/l tryptone, 5 g/l yeast extract and 5 g/l NaCl (all supplied by Nacalai Tesque, Kyoto, Japan)) supplemented with 100 μg/mL ampicillin. Yeast transformants and fusants were screened in synthetic dextrose (SD) medium (6.7 g/l yeast nitrogen base without amino acids (Difco Laboratories, Detroit, MI, USA) and 20 g/l glucose (Nacalai Tesque)) or synthetic PASC (SPASC) medium (6.7 g/l yeast nitrogen base without amino acids and 10 g/l PASC) supplemented with appropriate amino acids and nucleic acids. PASC was prepared from Avicel PH-101 (Fluka Chemie GmbH, Buchs, Switzerland) as amorphous cellulose [2].

Yeast cells were aerobically cultured in yeast/peptone/dextrose (YPD) medium (10 g/l yeast extract, 20 g/l peptone (Bacto-Peptone™; Difco Laboratories) and 20 g/l glucose) or molasses medium (5% v/v molasses, 0.5% (v/v) CSL (Sigma-Aldrich Japan, Tokyo, Japan) and 0.01% v/v antifoam SI (Wako Pure Chemical Industries, Ltd., Osaka, Japan)). The pH of molasses medium was adjusted to 5.0 by addition of sodium hydroxide. Aerobic culture proceeded in 1 liter flasks with 500 mL medium in a rotary shaker at 200 rpm. Ethanol fermentation proceeded in YP medium (10 g/l yeast extract and 20 g/l peptone (Bacto-Peptone™; Difco Laboratories)) supplemented with either 20 g/l PASC or with 100 g/l hot-water-pretreated (HWP) rice straw (Mitsubishi Heavy Industry, Tokyo, Japan).

### Hot-water pretreatment of rice straw

The water-insoluble fraction of HWP rice straw was washed twice with distilled water, dried at 80°C for 16 hours, ground to a particle size of approximately 0.5 mm using a laboratory disintegrator (Sansho Industry Co. Ltd., Osaka, Japan), and then used as the carbon source for ethanol fermentation. The composition of the prepared water-insoluble fraction, which was determined according to the procedure published by the National Renewable Energy Laboratory [18], was as follows: 44.8% w/w glucan, 0.3% w/w xylan and 0.8% w/w galactan, with low detectable amounts of arabinan and mannan.

### Yeast transformation and cocktail δ-integration

A cellulolytic enzyme expression-optimized strain that expressed the enzymes EG, CBH and BGL was first constructed by the cocktail δ-integration method, as described previously [8]. Briefly, identical amounts (about 20 μg of each plasmid) of three co-marked δ-integrative plasmids [19,20] (pδW-PGAGBGL, pδW-PGAGEG and pδW-PGAGCBH, which allow expression of BGL, EG and CBH, respectively, on the cell surface) were mixed and co-transformed into NBRC1440ΔHUWL. The transformants were spread on SPASC medium for selection, and several transformants were selected, based on colony size. Transformants with the highest cellulolytic activity were then selected by measuring their PASC-degradation activity. The selection cycle, consisting of co-transformation by three plasmids, screening on SPASC medium and measurement of PASC-degradation activity, was repeated three times using *TRP1*, *URA3 *and *HIS3 *as selection markers, and finally, one resultant transformant was selected and named 1440/cocδBEC3.

To screen mated diploid strains on SD medium without amino acids and nucleic acids, pRS405 was integrated into MT8-1/cocδBEC3 using a conventional integration method, as previously described [21]. The resultant transformant was designated MT8-1/cocδBEC3/LEU2.

### Diploidization by mating

The diploid strain MNII/cocδBEC3 was constructed by mating the haploid strains MT8-1/cocδBEC3/LEU2 and 1440/cocδBEC3, as described previously [13]. Briefly, both haploid strains were grown separately in liquid SD broth containing appropriate amino acids and nucleic acids for 24 hours at 30°C, harvested, spread together on SD plates supplemented with appropriate amino acids and nucleic acids, and incubated for a further 72 hours at 30°C. The strains were then replica-plated onto SD plates and incubated for 3 days at 30°C, then the resulting isolated single colonies were selected. The formation of diploid strain was confirmed by complementary amino acid markers and measurement of cell growth.

### Cell growth and PASC degradation activity

For measurements of cell growth and determination of PASC degradation activity, yeast cells were cultivated in YPD or molasses medium for 48 hours at 30°C (initial optical density (OD) at 600 nm was 0.05), collected by centrifugation at 3,000 × *g *for 5 minutes at 4°C and then washed twice with distilled water. Cell growth was determined by counting cell numbers microscopically in a hemocytometer (Bürker Türk) (NanoEnTek Inc., Gyeonggi, Korea) with appropriate dilution of cultures.

Washed cells were then added at a final concentration of 25 g wet cell/l to a 3 ml solution of 1 g/l PASC in 50 mmol/l sodium citrate buffer (pH 5.0) and 100 mmol/l methyl glyoxal (Nacalai Tesque), which was added to prevent assimilation of the produced glucose by yeast cells [22]. After the hydrolysis reaction was allowed to proceed at 50°C for 4 hours, the supernatant was collected by centrifugation for 5 minutes at 10,000 × *g *at 4°C to remove cells and debris, and the produced glucose concentration was measured (Glucose CII; Wako Pure Chemical Industries, Ltd., Osaka, Japan). One unit of PASC degradation activity was defined as the amount of enzyme producing 1 μmol/minute glucose at 50°C, pH 5.0.

### Quantification of transcription level of cellulolytic genes by real-time PCR

The transcription levels of each cellulolytic gene were quantified by real-time reverse transcription (RT)-PCR. Total RNA was isolated (RiboPure Yeast Kit; Ambion, Austin, TX, USA) from yeast cells cultivated in YPD medium for 48 hours at 30°C, and cDNA was then synthesized from total RNA (ReverTra Ace qPCR RT Kit; Toyobo, Osaka, Japan). Real-time RT-PCR using synthesized cDNA as a template was performed (ABI PRISM 7000 Sequence Detection System; Applied Biosystems, Foster City, CA, USA) with three sets of PCR primers (BGL 761F and BGL 858R, EGII 694F and EGII 774R and CBHII 571F and CBHII 653R [8]) and a SYBR green mix (Thunderbird SYBR qPCR Mix; Toyobo). Transcription levels of the three cellulolytic genes were normalized to the housekeeping gene *PGK1*, using a standard curve.

### Ethanol fermentation from PASC and rice straw

Yeast cells were precultured aerobically in YPD or molasses medium at 30°C for 48 hours, harvested by centrifugation at 1,000 × *g *for 5 minutes, and then washed twice with distilled water. The wet-cell weight was then determined by harvesting the washed cells by centrifugation at 3,000 × *g *for 5 minutes and weighing the cell pellet (the estimated dry-cell weight for all strains was approximately 0.15 times that of the wet-cell weight). The cells were then resuspended in 20 mL YP medium containing 20 g/l PASC or 100 g/l HWP rice straw at an initial cell concentration adjusted to 200 g wet cell/l. Ethanol fermentation proceeded at 37°C for 72 hours with mild agitation in 100-ml closed bottles, each equipped with a siliconized tube and check valve (Sanplatec Corp., Osaka, Japan) for CO_2 _outlet. Ethanol concentration was determined using a gas chromatograph (model GC-2010; Shimadzu, Kyoto, Japan) equipped with a flame ionization detector and a Durabond Free Fatty Acid Phase (DB-FFAP) column (60 m × 0.25 mm internal diameter, 0.5 μm film thickness; Agilent Technologies, Palo Alto, CA, USA), using helium as the carrier gas. The injection volume and split ratio was adjusted to 1 μL and 1:50, respectively. The column temperature was programmed to increase from 40°C to 170°C with a linear gradient of 10°C/minute.

## Results

### Ethanol production from PASC by haploid yeast strains

**Figure 1:**
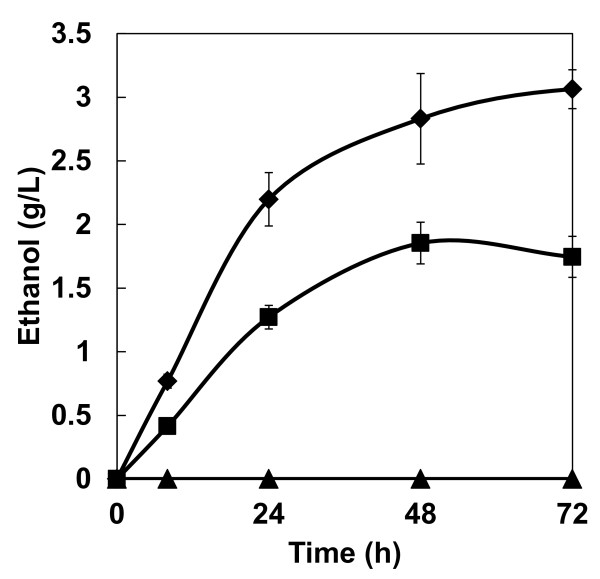
**Time course of ethanol production from PASC by haploid strains**. Triangles = MT8-1; squares = MT8-1/IBEC; diamonds = MT8-1/cocδBEC3. Data are averages from three independent experiments (error bars represent SE).

Ethanol productivity from PASC was first evaluated by performing fermentations with haploid *S. cerevisiae *strains that were previously engineered through either a conventional method or the cocktail δ-integration method to express the cellulases BGL, EG and CBH. The cellulolytic enzyme expression-optimized strain MT8-1/cocδBEC3 exhibited the highest ethanol productivity (Figure 1). The maximum ethanol production by MT8-1/cocδBEC3 from 20 g/l PASC reached 3.1 g/l in 72 hours, which was 1.6 times greater that of the conventionally integrated strain MT8-1/IBEC (1.9 g/l). The final ethanol yield by MT8-1/cocδBEC3 and MT8-1/IBEC from PASC after 72 hours of fermentation was 30% and 19% of the theoretical yield, respectively. Notably, wild-type strain MT8-1 did not produce detectable amounts of ethanol from PASC.

### Construction of a diploid yeast strain

To improve the cellulolytic activity of the recombinant haploid strain MT8-1/cocδBEC3 (*MAT*a, *ade*, *leu2*), the diploid strain MNII/cocδBEC3 (*MAT*a/*α*) was constructed by mating the haploid cellulolytic enzyme expression-optimized strain MT8-1/cocδBEC3 (which was previously constructed by the cocktail δ-integration [8]) with strain 1440/cocδBEC3 (*MATα leu2*).

### Cellulolytic enzyme expression ratio

**Figure 2:**
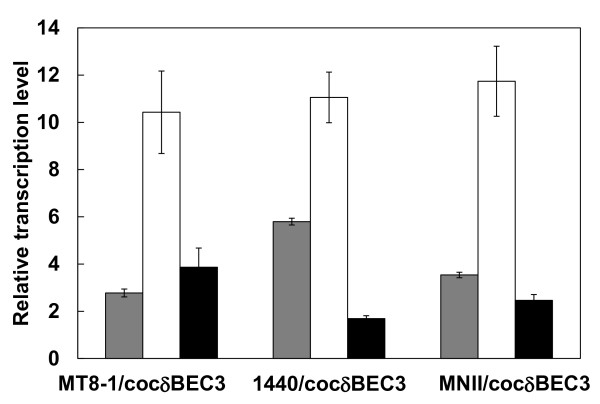
**Transcription levels of cellulolytic enzymes of haploid strain MT8-1/cocδBEC3, 1440/cocδBEC3 and diploid strain MNII/cocδBEC3**. Gray bar = β-glucosidase; white bar = endoglucanase; black bar = cellobiohydrolase. Data are averages from six independent experiments (error bars represent SE).

To evaluate the cellulolytic enzyme expression ratios of the haploid and diploid strains, transcription levels of the three cellulolytic enzymes were quantified by real-time RT-PCR (Figure 2). Although the transcription level ratios of the three types of cellulases were nearly identical in strains MT8-1/cocδBEC3, 1440/cocδBEC3 and MNII/cocδBEC3, the transcription level of EG was markedly higher than that of the other two enzymes.

### Cell growth and PASC degradation activity of haploid and diploid strains after cultivation in YPD and molasses medium

To examine differences in growth profiles and fermentation ability between haploid and diploid strains, cell growth and PASC degradation activity were evaluated after cultivation in YPD or molasses medium. Both the haploid strain MT8-1/cocδBEC3 and the diploid strain MNII/cocδBEC3 had higher cell growth in YPD medium (5.3 and 20.0 × 10^7 ^cells/mL, respectively) than in molasses medium (1.4 and 5.3 × 10^7 ^cells/mL, respectively) (Table 2)

**Table 2:** PASC degradation activity and cell growth of haploid and diploid strains

Medium	Strain	PASC degradation activity (mU/g wet cell)	Cell growth (×10^7 ^cells/mL)
YPD	MT8-1/cocδBEC3	180.1 ± 5.7	5.3 ± 0.15
	MNII/cocδBEC3	234.1 ± 7.2	20.0 ± 0.51
			
Molasses	MT8-1/cocδBEC3	63.5 ± 6.2	1.4 ± 0.08
	MNII/cocδBEC3	381.4 ± 6.6	5.3 ± 0.13

When cells of the two strains cultured in YPD and molasses medium were subjected to PASC hydrolysis reactions, the haploid strain MT8-1/cocδBEC3 had higher PASC degradation activity when precultured in YPD medium (180.1 mU/g wet cell) than in molasses medium (63.5 mU/g wet cell), whereas the diploid strain MNII/cocδBEC3 had lower PASC degradation activity when precultured in YPD medium (234.1 mU/g wet cell) than in molasses medium (381.4 mU/g wet cell). Despite this difference, the diploid strain MNII/cocδBEC3 had higher cell growth and PASC degradation activity than the haploid strain MT8-1/cocδBEC3, regardless of the medium used.

### Ethanol production from cellulose by the diploid yeast strain

**Figure 3:**
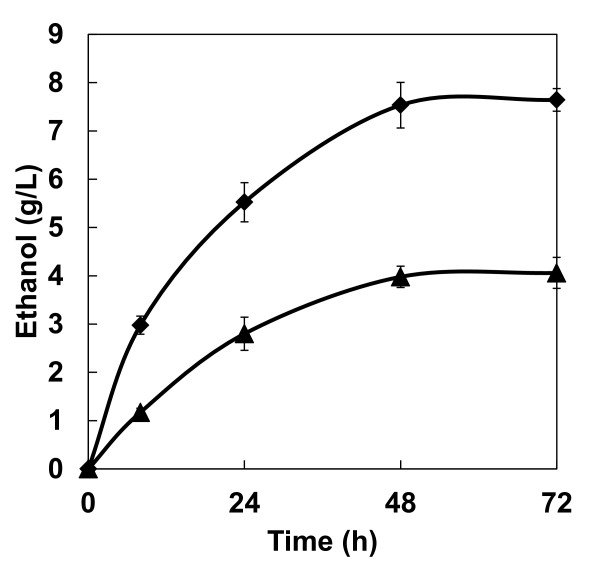
**Time course of ethanol production from PASC by diploid strain MNII/cocδBEC3**. Triangles = precultured using YPD; diamonds = precultured using molasses medium. Data are averages from three independent experiments (error bars represent SE).

Finally, to evaluate ethanol production by the diploid strain MNII/cocδBEC3, ethanol fermentations from PASC and pretreated rice straw were performed with cells precultured in either YPD or molasses medium (Figure 3). After 72 hours of fermentation at 37°C, maximum ethanol production from PASC by the diploid strain prepared using YPD or molasses medium reached 4.1 and 7.6 g/l, respectively, representing an increase of approximately twofold for cells precultured with molasses as a carbon source. The final ethanol yields from the initial cellulose content reached 40% and 75% of the theoretical yield (of 0.51 g ethanol/g glucose) in 72 hours for the diploid strain precultured in YPD and molasses medium, respectively.

**Figure 4:**
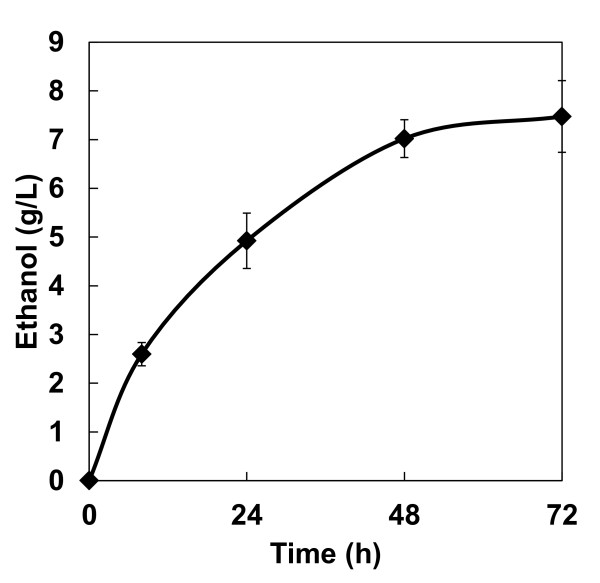
**Time course of ethanol production from pretreated rice straw by diploid strain MNII/cocδBEC3 precultured in molasses medium**. Data are averages from three independent experiments (error bars represent SE).

Figure 4 shows the time course of ethanol fermentation from rice straw by the diploid strain prepared using molasses medium. The maximum ethanol concentration and ethanol yield from the initial glucan reached 7.5 g/l and 33% of the theoretical yield in 72 hours, respectively.

## Discussion

In this study, we developed a cellulolytic enzyme expression-optimized diploid *S. cerevisiae *strain that was able to produce ethanol directly from PASC and pretreated rice straw with relatively high yields.

The expression-optimized MT8-1/cocδBEC3 strain had a PASC degradation activity (75 mU/g wet cell) that was approximately sixfold higher than that of the non-optimized MT8-1/IBEC3 strain (12 mU/g wet cell) [8], corresponding to a twofold higher ethanol productivity (Figure 1). These results clearly show that optimization of cellulolytic enzyme expression is effective in enhancing ethanol production from PASC and in improving PASC degradation ability; however, the total ethanol yield remained low. Therefore, to improve PASC degradation activity and increase ethanol production, we constructed a cellulolytic enzyme-expressing diploid strain with optimized cellulase expression.

PASC degradation activity was improved by diploidization (Table 2), which might have been due to the increased copy number of cellulase genes integrated into the genome [14]. Notably, in this cellulolytic enzyme expression-optimized diploid strain, MNII/cocδBEC, the transcription level of the EG gene was markedly higher than that of the other two integrated cellulase genes, BGL and CBH (Figure 2). This result correlates well with the observation that during the optimization process, the EG gene preferentially integrates into yeast genomes with high copy number compared with the BGL and CBH genes [8]. In addition, the transcription levels of the three cellulolytic genes in the haploid host strains MT8-1/cocδBEC3 and 1440/cocδBEC3 were nearly identical to those of MNII/cocδBEC3 (Figure 2), suggesting that total amount of gene expression could be improved by diploidization if the the optimized gene expression of the parental strains were retained. Hence, performing diploidization after optimization of multigene expression could be an effective strategy for constructing cellulose degrading yeast.

To reduce yeast preparation costs for industrial applications, we evaluated the cell growth of the haploid and diploid strains using molasses medium (Table 2). One advantage of the cocktail δ-integration method is that cultivation of recombinant strains under non-selective conditions is possible, because foreign genes integrated into the genome are stably maintained [14].The cell growth of both MT8-1/cocδBEC3 and MNII/cocδBEC3 in molasses medium (containing CSL as the nutrient source) was lower than in YPD medium. Although cell growth of MT8-1/cocδBEC3 in molasses medium was improved slightly by supplementation with essential amino acids, it was still twofold lower than YPD medium (data not shown). Thus, depression of cell growth was probably not caused by amino acid scarcity but rather by the nutrient richness of the YPD medium and susceptibility of the strain to inhibitory factors in molasses and/or CSL [23-26]. Surprisingly, however, the PASC degradation activity of MNII/cocδBEC3 after precultivation in molasses medium was significantly higher compared with that after growth in YPD medium. One possible explanation is that metal ions present in molasses and/or CSL improved the stability of the cellulolytic enzymes [27-29].

As expected from the PASC hydrolysis reaction results, high ethanol production and yield from PASC was achieved using the diploid strain prepared in molasses medium (Figure 3). When we compared our results with those from different cellulase-expression systems of *S. cerevisiae *published previously (Table 3), our diploid strain clearly achieved the highest ethanol production and yields. In addition to these promising findings, the cellulolytic enzyme-expressing diploid yeast strain was also able to produce ethanol from pretreated rice straw (Figure 4). Although the ethanol production rate from rice straw was nearly identical to that from PASC, the ethanol yield from rice straw was relatively low. This result suggests that highly crystalline regions of cellulose in rice straw were not effectively degraded, thus reducing these regions by improving the efficiency of pretreatment or further optimizing cellulolytic enzyme-expression ratios in recombinant diploid yeast may lead to improved bioethanol yields from agricultural waste biomass. Using the cocktail δ-integration method, cellulase expression in yeast could be optimized for degradation of rice straw [8]. Furthermore, hemicelluloses and a lignin matrix surrounding cellulose would also prevent effective degradation of lignocellulose [30]; however, these could be removed by pretreatment or with use of additional exogenously expressed enzymes.

**Table 3:** Comparison of ethanol productivity from PASC for several cellulase-expression systems in *S. cerevisiae*

Strain	Cellulase expression	Initial PASC concentration, g/l	Maximum ethanol production, g/l	Maximum ethanol reaching time, hours	Ethanol yield of the theoretical yield from initial cellulose, %^1^	Time and effort	Reference
*S. cerevisiae*^2^	Cell surface display	10	2.9	40	57	Triple yeast transformation	[3]
*S. cerevisiae*	Secretion	10	1.0	192	20	Single yeast transformation	[2]
*S. cerevisiae *and *E. coli*^3^	Minicellulosome constructed by *E. coli *produced cellulases	10	3.5	48	69	Single yeast transformation, cellulase production by *E*. *coli*, and immobilization	[4]
*S. cerevisiae*	Minicellulosome	10	1.8	70	35	Double yeast transformation	[5]
*S. cerevisiae*	Cell surface display in optimum ratio	20	3.1	72	30	Triple yeast transformation	This study
*S. cerevisiae*	Cell surface display in optimum ratio with diploidization	20	7.6	72	75	Triple yeast transformation and diploidization	This study

^1^Calculated from each result.

^2^*Saccharomyces cerevisiae*.

^3^*Escherichia coli*.

## Conclusions

We have developed a cellulolytic enzyme expression-optimized diploid yeast strain that is capable of directly fermenting from cellulosic materials. Although this is a proof-of-concept study, it is, to our knowledge, the first report of ethanol production from agricultural waste biomass using a recombinant cellulolytic enzyme-expressing diploid yeast without the addition of exogenous enzymes. In addition, the diploid strain can be cultivated in inexpensive industrial medium while retaining high PASC degradation activity. Multigene expression optimization in yeast using our cocktail δ-integration method could be used for improving the efficiency of ethanol production from various types of readily available lignocellulosic biomass.

## Competing interests

The authors declare that they have no competing interests.

## Authors' contributions

RY designed and performed the experiments. NT performed the experiments. RY and TT wrote the manuscript. CO, HF and AK supervised the research, and helped to draft the manuscript. All authors read and approved the final manuscript.
